# Real time remote symptom monitoring during chemotherapy for cancer: European multicentre randomised controlled trial (eSMART)

**DOI:** 10.1136/bmj.n1647

**Published:** 2021-07-21

**Authors:** Roma Maguire, Lisa McCann, Grigorios Kotronoulas, Nora Kearney, Emma Ream, Jo Armes, Elisabeth Patiraki, Eileen Furlong, Patricia Fox, Alexander Gaiger, Paul McCrone, Geir Berg, Christine Miaskowski, Antonella Cardone, Dawn Orr, Adrian Flowerday, Stylianos Katsaragakis, Andrew Darley, Simone Lubowitzki, Jenny Harris, Simon Skene, Morven Miller, Margaret Moore, Liane Lewis, Nicosha DeSouza, Peter T Donnan

**Affiliations:** 1 Computer and Information Sciences, University of Strathclyde, Glasgow, UK; 2 School of Medicine, Dentistry and Nursing, University of Glasgow, Glasgow, UK; 3 Leven, UK; 4 University of Surrey, School of Health Sciences, Guildford, UK; 5 National and Kapodistrian University of Athens School of Health Sciences, Athens, Greece; 6 School of Nursing, Midwifery and Health Systems, University College Dublin, Dublin, Ireland; 7 Department of Internal Medicine 1, Division of Hematology and Hemostaseology, Medical University of Vienna, Vienna, Austria; 8 Department of Health Services and Population Research, King’s College London Institute of Psychiatry Psychology and Neuroscience, London, UK; 9 Department of Health Sciences, NTNU, Gjøvik, Norway; 10 University of California San Francisco, San Francisco, CA, USA; 11 European Cancer Patient Coalition, Brussels, Belgium; 12 NHS 24, Glasgow, UK; 13 Docobo Limited, Leatherhead, UK; 14 School of Medicine, University College Dublin, Dublin, Ireland; 15 Surrey Clinical Trials Unit, University of Surrey, Guildford, UK; 16 Johnson and Johnson Medical, Norderstedt, Germany; 17 Population Health and Genomics, Medical School, University of Dundee, Dundee, UK

## Abstract

**Objective:**

To evaluate effects of remote monitoring of adjuvant chemotherapy related side effects via the Advanced Symptom Management System (ASyMS) on symptom burden, quality of life, supportive care needs, anxiety, self-efficacy, and work limitations.

**Design:**

Multicentre, repeated measures, parallel group, evaluator masked, stratified randomised controlled trial.

**Setting:**

Twelve cancer centres in Austria, Greece, Norway, Republic of Ireland, and UK.

**Participants:**

829 patients with non-metastatic breast cancer, colorectal cancer, Hodgkin’s disease, or non-Hodgkin’s lymphoma receiving first line adjuvant chemotherapy or chemotherapy for the first time in five years.

**Intervention:**

Patients were randomised to ASyMS (intervention; n=415) or standard care (control; n=414) over six cycles of chemotherapy.

**Main outcome measures:**

The primary outcome was symptom burden (Memorial Symptom Assessment Scale; MSAS). Secondary outcomes were health related quality of life (Functional Assessment of Cancer Therapy—General; FACT-G), Supportive Care Needs Survey Short-Form (SCNS-SF34), State-Trait Anxiety Inventory—Revised (STAI-R), Communication and Attitudinal Self-Efficacy scale for cancer (CASE-Cancer), and work limitations questionnaire (WLQ).

**Results:**

For the intervention group, symptom burden remained at pre-chemotherapy treatment levels, whereas controls reported an increase from cycle 1 onwards (least squares absolute mean difference −0.15, 95% confidence interval −0.19 to −0.12; P<0.001; Cohen’s D effect size=0.5). Analysis of MSAS sub-domains indicated significant reductions in favour of ASyMS for global distress index (−0.21, −0.27 to −0.16; P<0.001), psychological symptoms (−0.16, −0.23 to −0.10; P<0.001), and physical symptoms (−0.21, −0.26 to −0.17; P<0.001). FACT-G scores were higher in the intervention group across all cycles (mean difference 4.06, 95% confidence interval 2.65 to 5.46; P<0.001), whereas mean scores for STAI-R trait (−1.15, −1.90 to −0.41; P=0.003) and STAI-R state anxiety (−1.13, −2.06 to −0.20; P=0.02) were lower. CASE-Cancer scores were higher in the intervention group (mean difference 0.81, 0.19 to 1.43; P=0.01), and most SCNS-SF34 domains were lower, including sexuality needs (−1.56, −3.11 to −0.01; P<0.05), patient care and support needs (−1.74, −3.31 to −0.16; P=0.03), and physical and daily living needs (−2.8, −5.0 to −0.6; P=0.01). Other SCNS-SF34 domains and WLQ were not significantly different. Safety of ASyMS was satisfactory. Neutropenic events were higher in the intervention group.

**Conclusions:**

Significant reduction in symptom burden supports the use of ASyMS for remote symptom monitoring in cancer care. A “medium” Cohen’s effect size of 0.5 showed a sizable, positive clinical effect of ASyMS on patients’ symptom experiences. Remote monitoring systems will be vital for future services, particularly with blended models of care delivery arising from the covid-19 pandemic.

**Trial registration:**

Clinicaltrials.gov NCT02356081.

## Introduction

Despite guidance for patients and professionals on managing chemotherapy, associated symptoms are often poorly controlled.^1^ ^2^ Undertreatment of symptoms results in poorer adherence to treatment, impaired health related quality of life, increased health service use, and mortality.^3^ ^4^ ^5^ Current symptom assessment mechanisms rely on patients recognising that symptoms are severe enough to warrant reporting. Uncertainty, delayed reporting, and inability to access 24 hour services frequently lead to toxicities not being recognised sufficiently quickly, placing patients’ safety at risk.^6^ ^7^ Solutions are needed to expedite this process to improve patients’ outcomes and reduce costs.

Emergence of connected health has driven proliferation of remote monitoring solutions to support patients in community settings.^8^ ^9^ These seem to be beneficial for people receiving chemotherapy, as they can circumnavigate the need for retrospective assessment, a process believed to “provide a weaker insight into actual symptom burden” as a result of recall bias.^5^ Such systems also enable patients’ data to be relayed to clinicians within minutes, enabling proactive symptom management. Previous research on remote monitoring systems in the chemotherapy context highlights their benefits for health related quality of life, symptom alleviation, avoiding unscheduled hospital admissions, survival, and cost effectiveness.^10^ ^11^ ^12^ ^13^ ^14^ However, this evidence is largely derived from trials, of short duration and from a single site/country, in people with advanced disease and without health economic evaluation.^15^ ^16^ Evidence to inform scale-up across multiple countries and enable benefits to be realised globally is also limited.^17^

eSMART aimed to provide definitive high quality evidence on large scale benefits of remote monitoring in the management of toxicities of adjuvant and first line chemotherapy. eSMART was underpinned by earlier work, reflecting the Medical Research Council’s framework for designing and evaluating complex interventions.^18^ ^19^ This showed the feasibility and acceptability of the Advanced Symptom Management System (ASyMS).^20^ ^21^ ^22^ ^23^ The trial hypothesis was that, compared with standard care, ASyMS would lead to reductions in symptom burden, supportive care needs, anxiety, and work limitations and improvements in health related quality of life and self-care/self-efficacy during chemotherapy treatment in patients with breast cancer, colorectal cancer, Hodgkin’s disease, or non-Hodgkin’s lymphoma.

## Methods

We conducted a multicentre, parallel group randomised controlled trial, using 1:1 allocation of study participants, between 31 March 2016 and 31 March 2019 (recruitment closed 14 December 2018). No significant changes were made to the methods during the trial,^24^ which was registered on Clinicaltrials.gov (NCT02356081). A data monitoring committee monitored progress and patient safety and met on average six monthly. The study is reported in accordance with the CONSORT statement for randomised controlled trials.

### Setting and participants

Clinical or research staff from 12 cancer centres across Austria, Greece, Ireland, Norway, and the UK recruited participants. Participants attended for an enrolment visit before their first chemotherapy cycle, and written informed consent was obtained at this visit.

Eligible patients were aged ≥18 years; diagnosed as having breast cancer, colorectal cancer, Hodgkin’s disease, or non-Hodgkin’s lymphoma; scheduled to receive at least three cycles of two, three, or four weekly first line adjuvant chemotherapy or chemotherapy for the first time in five years; physically and psychologically fit to participate; and able to understand and communicate in the language of the country where recruited. The inclusion criteria were intentionally narrow to ensure that only patients treated with curative intent were recruited.

Participants were ineligible if they had distant metastasis (breast or colorectal cancer) or B symptoms (Hodgkin’s disease/non-Hodgkin’s lymphoma), were receiving concurrent radiotherapy or weekly chemotherapy (as timeframes covered by outcome measures were incompatible with weekly administration), had been diagnosed as having cancer or received chemotherapy within the previous five years, or were unable to provide informed consent.

Patients were initially recruited for the full duration of their chemotherapy. However, after five months, this was amended to participation for a maximum of six cycles to support recruitment and study timelines. This change was supported by previous ASyMS pilot work (unpublished) and literature showing that chemotherapy related symptoms are greatest during initial cycles of treatment.^25^ Consequently, participants used ASyMS when they were likely to gain most benefit.

### Intervention

**Fig 1:**
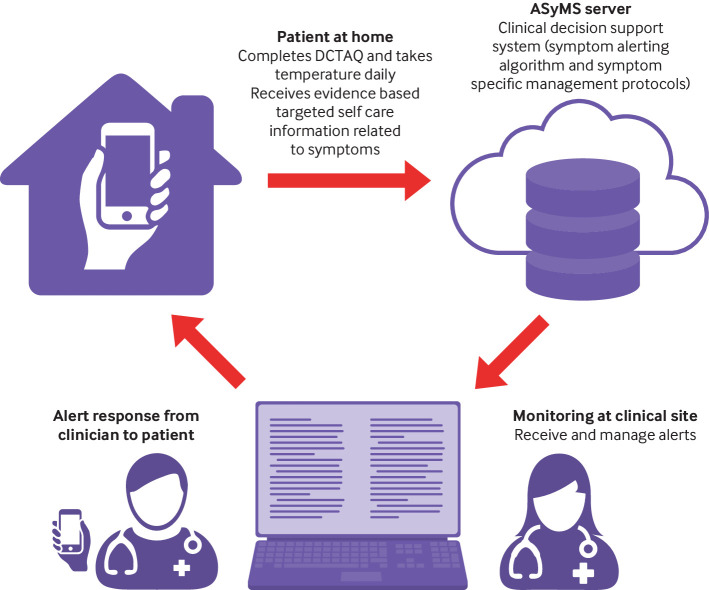
Advanced Symptom Management System (ASyMS) intervention. DCTAQ=Daily Chemotherapy Toxicity Self-Assessment Questionnaire

ASyMS (fig 1) provides real time, 24 hour monitoring and management of chemotherapy toxicity. Patients use ASyMS to complete a validated self-reported questionnaire (Daily Chemotherapy Toxicity Self-Assessment Questionnaire (DCTAQ)) that assesses 10 symptoms (nausea, vomiting, diarrhoea, constipation, mucositis, paraesthesia, sore hands/feet, flu-like symptoms/infection, tiredness, pain) and up to six additional symptoms.^26^ Patients are also asked to take and enter their body temperature by using a digital thermometer. Patients can access tailored, evidence based self-care advice (adapted from Macmillan Cancer Support resources) on ASyMS at any time.

ASyMS was translated into native languages (patient/clinician handsets, website, manuals). Patients completed the DCTAQ daily and whenever they felt unwell. “Real time” information was sent via a secure connection to the ASyMS server hosted by Docobo (https://www.docobo.co.uk/). After a review of local, national, and European best practice symptom management guidelines, and based on previous ASyMS studies, an evidence based clinical decision system, incorporating a symptom alerting algorithm and symptom specific management protocols, was developed for ASyMS and verified by cancer experts from each clinical site before use.^27^

Symptom data were automatically evaluated in ASyMS and, when necessary, generated two types of alerts to hospital clinicians: amber, for persistent mild-moderate symptoms for which early intervention could prevent progression; and red, for chemotherapy emergencies such as neutropenic sepsis. Required response times were eight hours for amber alerts and 30 minutes for red alerts. When an alert was generated before an earlier alert had been dealt with (for example, if the patient completed a subsequent DCTAQ because symptoms had worsened), the alerts were linked.

**Fig 2:**
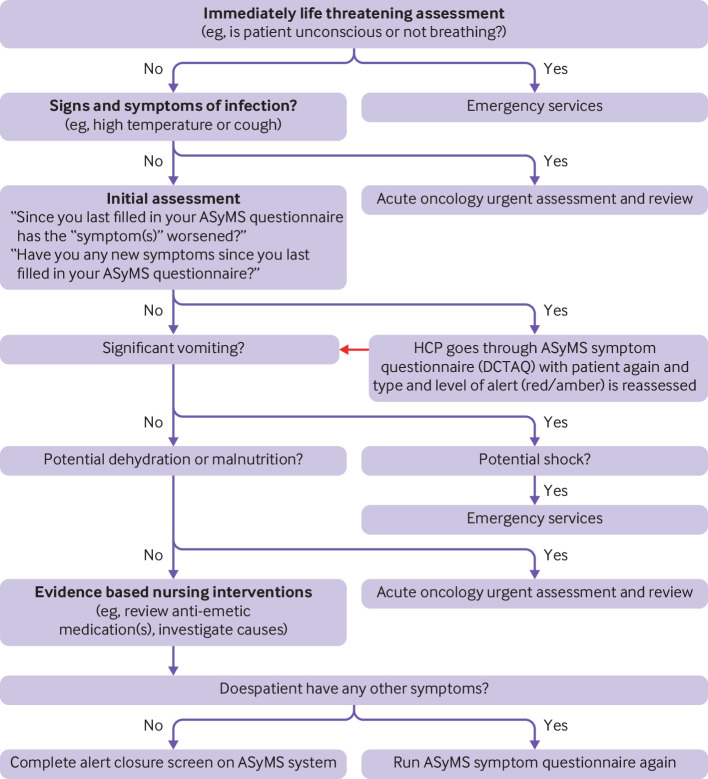
Overview of example symptom management protocol (nausea and vomiting) on Advanced Symptom Management System (ASyMS). DCTAQ=Daily Chemotherapy Toxicity Self-Assessment Questionnaire; HCP=healthcare professional

Clinicians received alerts on dedicated handsets. For red alerts, clinicians were required to review patients’ symptom reports on ASyMS web pages and contact the patient for further review. For amber alerts, although viewing of reports was required, clinicians had the option of contacting patients. This discretionary element was incorporated following piloting in which clinicians indicated that in instances when a patient had generated an amber alert and had received self-care advice the previous day, contacting the patient the next day was often not necessary if the same alert was triggered again. During calls with patients, clinicians worked through evidence based clinical decision support systems embedded within ASyMS (fig 2) to inform symptom management interventions. On closing the alert, clinicians detailed actions taken—for example, self-care or acute oncology referral—and could select more than one action.

Participants in the intervention group used ASyMS for up to six cycles. They were trained by researchers/clinicians at each site on its use before starting chemotherapy. Participants in the control group received care as usual at their cancer centre and were advised to contact their clinician(s) through standard mechanisms (usually telephone triage) if they developed symptoms. This group received clinical input in line with each local site’s standard advice (verbal and written) on chemotherapy related symptoms and self-care.

### Outcome measures

All participants completed patient reported outcome measures recording primary and secondary outcomes at baseline (enrolment, before randomisation, before chemotherapy) and before subsequent cycles. Patient reported outcome measures were collected in the cancer centre, via a tablet or secure weblink, coinciding with chemotherapy visits.

We collected data on adverse events and hospital admissions by reviewing case notes. Specifically, we defined a “neutropenic sepsis event” as development of fever (oral temperature >38.5°C or two consecutive readings of >38.0°C for two hours) and other signs of generalised, whole body infection in a patient with neutropenia (an absolute neutrophil count <0.5×10^9^/L, or <1.0×10^9^/L and “falling”). We also investigated hypothermia in the presence of neutropenia as a sign of neutropenic sepsis.^28^ Device related incidents were monitored during the trial according to European Commission DG Enterprise and Industry guidelines on a medical devices vigilance system.^29^

The primary outcome measure was the Memorial Symptom Assessment Scale (MSAS),^30^ which measures 32 physical and psychological cancer related symptoms. Respondents reported on the occurrence, frequency, and severity of, and the distress associated with, each symptom over the preceding week. We calculated total MSAS score, representing symptom burden, by averaging items (that is, a potential range of 0-4). If >13% of items were missing, we treated the score as missing.^30^ Additionally, we calculated three subscale scores (global distress index, physical score, and psychological score) and treated them as secondary outcomes. The reliability and validity of MSAS are well established; in this study Cronbach’s α for total MSAS and MSAS physical, MSAS psychological, and MSAS global distress index subscales was 0.87, 0.82, 0.77, and 0.83, respectively.

Full details of the secondary outcomes measures are published in the eSMART study protocol.^24^ Briefly they included Functional Assessment of Cancer Therapy—General (FACT-G),^31^ assessing health related quality of life and four domains of wellbeing (physical, social/family, emotional, and functional); Supportive Care Needs Survey Short-Form (SCNS-SF34),^32^ measuring supportive care needs in five domains (health system and information, psychological needs, physical and daily living, patient care and support, and sexual related); State-Trait Anxiety Inventory—Revised (STAI-R),^33^ measuring two types of anxiety (state (about an event) and trait (anxiety as a personal characteristic)); Communication and Attitudinal Self-Efficacy scale for cancer (CASE-Cancer),^34^ assessing cancer patients’ confidence and ability to engage in their care plus three dimensions (maintaining a positive attitude, understanding and participating in care, and seeking and obtaining information); and Work Limitations Questionnaire (WLQ),^35^ a 25 item scale corresponding to four domains (time management, physical demands, mental/interpersonal demands, and output demands), which was completed only by participants who were working.

Additional secondary outcomes assessed the effectiveness of ASyMS one year after the randomised controlled trial, evaluated the cost effectiveness of ASyMS, and assessed changes in clinical practice. These secondary outcomes will be reported in forthcoming publications.

### Adherence of patients and clinicians to intervention

We measured patients’ adherence by calculating compliance with DCTAQ for each patient, defined as the number of days on which at least one DCTAQ was completed divided by the number of days when a DCTAQ was available for completion. Participants were instructed to complete the DCTAQ daily but could complete additional DCTAQs any time they felt unwell. We calculated adherence rates by using the number of days on which DCTAQs were completed, rather than the number of DCTAQs completed, as participants may have completed more than one per day, over-inflating adherence rates. We calculated clinical adherence by measuring time taken for clinicians to first view ASyMS alerts on the server and calculating the percentage of linked amber and red alerts managed within the specified timeframes.

### Randomisation and blinding

Surrey Clinical Trials Unit randomised patients remotely and independently using the Promasys system after patient consent and completion of baseline patient reported outcome measures and before the first chemotherapy cycle. Randomisation was stratified by site and cancer type. Research staff accessed the Promasys system remotely and completed a web based electronic case report form for the participant, and the allocation was assigned automatically. Random allocations were programmed by statisticians at the clinical trials unit using proc plan in SAS and run by staff independent of the trial team to ensure concealment of allocation. The nature of the intervention meant that blinding of patients was not possible, but patients were blinded to study hypotheses. Blinding of evaluators was achieved, as participants’ allocation was concealed from the statistical analysis team.

### Sample size

During the planning of the trial, only limited literature was available, and it focused on the short version of MSAS (MSAS-SF) and on single patient groups followed over time. Chang et al reported clinically significant differences of 0.20-0.66 for a change in total MSAS-SF scores from baseline to one week later in patients with the worst cancer pain,^36^ and Dapueto et al reported absolute difference in global distress index of 0.38 and a Cohen’s effect size of 0.6 for a change in MSAS-SF global distress index scores between the first cycle and third month of adjuvant chemotherapy.^37^ Given the lack of data on MSAS from intervention studies, a priori we considered a small to moderate Cohen’s effect size of 0.25 to be a clinically significant difference in total MSAS scores. We then based sample size estimation based on differences in total MSAS scores between intervention and control groups of 1.45–1.30=0.15 from a previous randomised controlled trial.^38^ For a difference in total MSAS scores of 0.15 (SD 0.6), with baseline and four repeated measures after enrolment, we needed a sample of 776 participants (110 participants with Hodgkin’s disease or non-Hodgkin’s lymphoma, 333 participants with breast cancer, and 333 patients with colorectal cancer) to provide 90% power for a two sided hypothesis test at the 5% significance level. Allowing for 30% attrition, we increased the total study sample size to 1108. By February 2017 the attrition rate was lower than anticipated (10%); consequently, we reduced the sample size to 862.

### Data analysis

We did an intention to treat analysis. We present study outcomes as means and standard deviations or percentages and denominators. Transformations were needed when distributions were non-normal, and we present medians and ranges. We present enrolment characteristics for the evaluable intention to treat sample. The primary outcome of total MSAS score is continuous, and we assessed it in a repeated measures analysis using linear and non-linear mixed models. We used a mixed model repeated measures analysis that uses all available data, assuming data are missing at random, throughout the analyses. Many participants rated their symptoms as not present (that is, 0), so the mixed model was implemented with a zero inflated γ distribution and identity link to provide results on the original scale.

Analyses tested the between group difference in means for the primary outcome—total symptom burden (total MSAS)—during chemotherapy for up to six cycles. We tested the primary hypothesis (reduced symptom burden in the intervention group over six cycles) through the regression parameter for the intervention versus control group, adjusting for baseline MSAS. We also adjusted for pre-specified variables: cancer type (breast cancer, colorectal cancer, Hodgkin’s disease, non-Hodgkin’s lymphoma), age (years), sex (male, female), number of comorbidities (0, 1-4, ≥5), and country (Austria, Greece, Republic of Ireland, Norway, and UK). We did pre-specified subgroup analyses by fitting trial arm by subgroup interaction parameters. If this test was significant at the 5% level, we estimated results separately for different subgroups. We used SAS software (version 9.4) and R 3.5 for all statistical analyses. We took a two sided P value of <0.05 as significant for all analyses unless otherwise specified.

### Patient and public involvement

The European Cancer Patient Coalition was a partner in eSMART and advised throughout the study. It was involved in setting research priorities, defining research questions and outcome measures, and providing input into recruitment and data collection and dissemination.

## Results

### Recruitment

**Fig 3:**
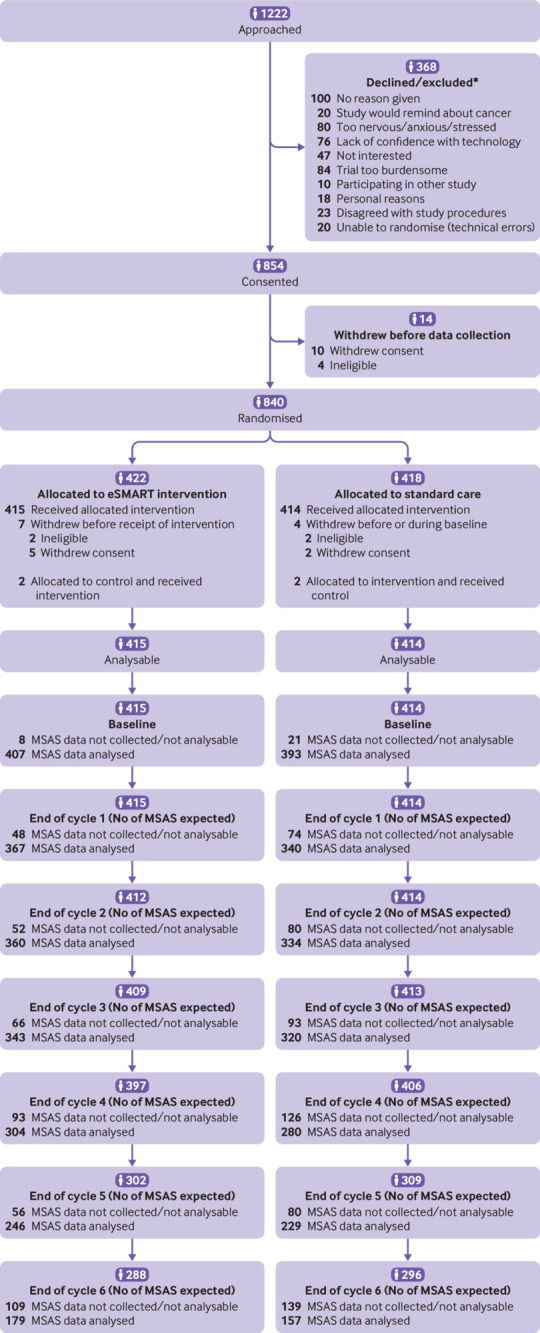
CONSORT diagram for eSMART. *Some people reported more than one reason for not wanting to take part, so total number of reasons exceeds number declined/excluded

We recruited 840 patients; seven participants in the intervention group and four in the control group were ineligible after randomisation or immediately withdrew consent, leaving 415 and 414 analysable (fig 3). Not all the patients recruited participated for six cycles of chemotherapy. Reasons varied but included prescription of less than six cycles, discontinuation of chemotherapy, withdrawal, and death. Most participants (n=723; 87.2%) completed four to six cycles. Attrition was low at 8.2% (n=34) in the intervention group and 9.2% (n=38) in the control group. Figure 3 provides details for the primary outcome, noting the number expected, the number not collected/not analysable, and the number analysed.

### Enrolment characteristics

Randomisation achieved a good balance between study arms for age, sex, country, cancer type, employment, and educational status (more in the intervention group reported a higher degree). Mean age was 52.4 (SD 12.2) years, and 81.8% were female. Breast cancer was most common (71.4%), and most had early stage (0-II) disease (53.4%). The greatest numbers of participants were recruited from the UK (31.7%) and Greece (31.2%). Half had no comorbidities (table 1).

**Table 1:** Characteristics of participants at enrolment. Values are numbers (percentages) unless stated otherwise

Characteristics	All participants (n=829)	Intervention group (n=415)	Standard care group (n=414)
Mean (SD) age	52.4 (12.2)	51.9 (12.4)	52.9 (12.1)
Female sex	678 (81.8)	340 (81.9)	338 (81.6)
Marital status:			
Married	553 (66.7)	273 (65.8)	280 (67.6)
Single	129 (15.6)	68 (16.4)	61 (14.7)
Divorced	87 (10.5)	45 (10.8)	42 (10.1)
Widowed	38 (4.6)	14 (3.4)	24 (5.8)
Not known	22 (2.7)	15 (3.6)	7 (1.7)
Education:			
Primary	60 (7.2)	30 (7.2)	30 (7.2)
Secondary	318 (38.4)	141 (34.0)	177 (42.8)
University	439 (53.0)	235 (56.6)	204 (49.3)
Not known	12 (1.4)	9 (2.2)	3 (0.7)
Employment:			
Full time	375 (45.2)	192 (46.3)	183 (44.2)
Part time	109 (13.1)	53 (12.8)	56 (13.5)
Home maker	82 (9.9)	40 (9.6)	42 (10.1)
Unemployed	67 (8.1)	34 (8.2)	33 (8.0)
Retired	173 (20.9)	82 (19.8)	91 (22.0)
Rather not say	23 (2.8)	14 (3.4)	9 (2.2)
Smoking:			
Never	414 (49.9)	214 (51.6)	200 (48.3)
Ex-smoker	280 (33.8)	133 (32.0)	147 (35.5)
Not every day	38 (4.6)	16 (3.9)	22 (5.3)
Every day	97 (11.7)	52 (12.5)	45 (10.9)
Alcohol consumption:			
Every day	32 (3.9)	18 (4.3)	14 (3.4)
Occasionally	593 (71.5)	306 (73.7)	287 (69.3)
Never	204 (24.6)	91 (21.9)	113 (27.3)
Country:			
Austria	140 (16.9)	71 (17.1)	69 (16.7)
Greece	259 (31.2)	127 (30.6)	132 (31.9)
Ireland	135 (16.3)	68 (16.4)	67 (16.2)
Norway	32 (3.9)	16 (3.9)	16 (3.9)
UK	263 (31.7)	133 (32.0)	130 (31.4)
No of comorbidities:			
0	420 (50.7)	220 (53.0)	200 (48.3)
1-4	393 (47.4)	188 (45.3)	205 (49.5)
≥5	16 (1.9)	7 (1.7)	9 (2.2)
Staging*:			
Stage 0	3 (0.4)	2 (0.5)	1 (0.2)
Stage I	129 (15.6)	63 (15.2)	66 (15.9)
Stage II	310 (37.4)	154 (37.1)	156 (37.7)
Stage III	310 (37.4)	157 (37.8)	153 (37.0)
Stage IV†	21 (2.5)	10 (2.4)	11 (2.7)
Undefined‡	56 (6.8)	29 (7.0)	27 (6.5)
No of chemotherapy cycles:			
1	3 (0.4)	3 (0.7)	0
2	4 (0.5)	3 (0.7)	1 (0.2)
3	19 (2.3)	12 (2.9)	7 (1.7)
4	192 (23.2)	95 (22.9)	97 (23.4)
5	27 (3.3)	14 (3.4)	13 (3.1)
6	504 (60.8)	248 (59.8)	256 (61.8)
7§	10 (1.2)	8 (1.9)	2 (0.5)
8§	65 (7.8)	30 (7.2)	35 (8.5)
12§	5 (0.6)	2 (0.5)	3 (0.7)
Median (range) No of chemotherapy cycles	6 (1-12)	6 (1-12)	6 (2-12)

* TNM/UICC system for breast/colorectal cancer; Ann Arbor Staging System for haematological cancers.

† Not an exclusion criterion for participants with haematological cancer.

‡ Data not captured at clinical site before end of trial.

§ Until substantial amendment to study protocol was made to limit patients’ participation to ≤6 chemotherapy cycles, first 80 patients enrolled in trial participated until end of their prescribed chemotherapy protocol, and therefore show as having completed >6 cycles.

### Primary outcome

Table 2 shows mean total MSAS scores for the intervention and control groups at each assessment. In the intervention group, symptom burden remained at pre-chemotherapy treatment levels over all six chemotherapy cycles. By contrast, the control group reported an increase from cycle 1, which then slowly reduced over the five subsequent chemotherapy cycles. Using an identity link to give results on the original scale, the adjusted analysis least squares means on repeated measures were 0.36 in the intervention arm and 0.52 in the standard care arm, giving a difference of −0.15 (95% confidence interval −0.19 to-0.12; P<0.001) in favour of the intervention (table 3). This is equivalent to a Cohen’s effect size of 0.5.

**Table 2:** Descriptive summary of primary outcome – total Memorial Symptom Assessment Scale (MSAS) and sub-domains

	Intervention group				Standard care group		
	No	Mean (SD)	Median (range)		No	Mean (SD)	Median (range)
**Total MSAS**							
Baseline	407	0.35 (0.30)	0.27 (0-1.62)		393	0.39 (0.31)	0.32 (0-1.94)
Cycle 1	367	0.33 (0.27)	0.25 (0-1.35)		340	0.51 (0.42)	0.40 (0-2.50)
Cycle 2	360	0.35 (0.28)	0.28 (0-1.98)		334	0.53 (0.40)	0.44 (0-2.25)
Cycle 3	343	0.35 (0.31)	0.28 (0-1.45)		320	0.52 (0.44)	0.43 (0-3.24)
Cycle 4	304	0.37 (0.31)	0.31 (0-1.62)		280	0.53 (0.44)	0.45 (0-3.04)
Cycle 5	246	0.34 (0.29)	0.27 (0-1.45)		229	0.52 (0.41)	0.44 (0-2.22)
Cycle 6	179	0.37 (0.28)	0.30 (0-1.39)		157	0.48 (0.39)	0.38 (0-1.88)
**MSAS global distress index**							
Baseline	406	0.66 (0.55)	0.56 (0. 3.24)		393	0.73 (0.55)	0.60 (0-3.36)
Cycle 1	366	0.42 (0.47)	0.25 (0-2.36)		337	0.66 (0.62)	0.48 (0-3.14)
Cycle 2	361	0.42 (0.46)	0.28 (0-3.08)		334	0.67 (0.61)	0.51 (0-3.12)
Cycle 3	343	0.44 (0.49)	0.28 (0-2.46)		318	0.70 (0.64)	0.56 (0-3.48)
Cycle 4	304	0.46 (0.46)	0.32 (0-2.18)		277	0.69 (0.65)	0.56 (0-3.58)
Cycle 5	246	0.42 (0.49)	0.27 (0-2.40)		227	0.67 (0.57)	0.56 (0-2.68)
Cycle 6	179	0.44 (0.46)	0.32 (0-2.46)		157	0.62 (0.58)	0.44 (0-2.80)
**MSAS psychological**							
Baseline	400	0.90 (0.80)	0.67 (0-3.57)		388	1.00 (0.77)	0.84 (0-3.83)
Cycle 1	365	0.48 (0.60)	0.26 (0-3.08)		340	0.72 (0.74)	0.51 (0-3.90)
Cycle 2	359	0.46 (0.55)	0.31 (0-3.69)		334	0.68 (0.74)	0.46 (0-3.23)
Cycle 3	343	0.47 (0.61)	0.26 (0-3.07)		323	0.67 (0.74)	0.46 (0-3.74)
Cycle 4	304	0.51 (0.57)	0.31 (0-2.62)		280	0.69 (0.75)	0.46 (0-3.57)
Cycle 5	246	0.48 (0.64)	0.26 (0-3.59)		231	0.65 (0.69)	0.46 (0-3.46)
Cycle 6	179	0.52 (0.62)	0.31 (0-2.71)		157	0.64 (0.68)	0.51 (0-3.83)
**MSAS physical**							
Baseline	406	0.27 (0.35)	0.15 (0-2.10)		395	0.31 (0.36)	0.20 (0-1.97)
Cycle 1	365	0.27 (0.31)	0.17 (0-1.54)		338	0.51 (0.47)	0.38 (0-2.36)
Cycle 2	360	0.31 (0.37)	0.19 (0-2.19)		336	0.54 (0.46)	0.46 (0-2.66)
Cycle 3	344	0.34 (0.38)	0.24 (0-2.07)		319	0.56 (0.50)	0.48 (0-3.28)
Cycle 4	305	0.35 (0.36)	0.25 (0-2.01)		280	0.57 (0.51)	0.47 (0-2.97)
Cycle 5	247	0.32 (0.34)	0.20 (0-1.51)		226	0.56 (0.47)	0.43 (0-2.26)
Cycle 6	179	0.34 (0.33)	0.26 (0-1.71)		157	0.49 (0.45)	0.38 (0-2.01)

**Table 3:** Mixed model, repeated measures analysis of change from baseline using γ model

Variable	Adjusted* least squares mean (95% CI)			Adjusted* mean difference (95% CI)	
	Intervention	Standard care		Intervention *v* standard care	P value
Total MSAS†	0.36 (0.34 to 0.39)	0.52 (0.49 to 0.54)		−0.15 (−0.19 to −0.12)	<0.001
MSAS global distress index	0.46 (0.42 to 0.50)	0.67 (0.63 to 0.71)		−0.21 (−0.27 to −0.16)	<0.001
MSAS psychological	0.51 (0.46 to 0.55)	0.67 (0.63 to 0.72)		−0.16 (−0.23 to −0.10)	<0.001
MSAS physical	0.33 (0.30 to 0.36)	0.54 (0.51 to 0.58)		−0.21 (−0.26 to −0.17)	<0.001
FACT-G total	86.3 (85.3 to 87.3)	82.3 (81.3 to 83.3)		4.06 (2.65 to 5.46)	<0.001
FACT-G physical	23.4 (21.3 to 23.7)	21.6 (21.3 to 22.0)		1.75 (1.25 to 2.25)	<0.001
FACT-G emotional	20.4 (20.2 to 20.7)	19.9 (19.6 to 20.1)		−0.54 (−1.23 to 0.16)	0.13
FACT-G social	23.6 (23.2 to 23.9)	23.2 (22.8 to 23.5)		0.44 (−0.06 to 0.93)	0.08
FACT-G functional	19.1 (18.7 to 19.5)	17.5 (17.1 to 17.9)		1.61 (1.00 to 2.22)	<0.001
STAI-R trait	32.7 (32.2 to 33.3)	33.9 (33.4 to 34.4)		−1.15 (−1.90 to −0.41)	0.003
STAI-R state	31.9 (31.2 to 32.6)	33.0 (32.4 to 33.7)		−1.13 (−2.06 to −0.20)	0.02
CASE-Cancer	43.7 (43.3 to 44.2)	42.9 (42.3 to 43.4)		0.81 (0.19 to 1.43)	0.01
SCNS-SF34 psychological	23.2 (21.9 to 24.6)	24.4 (23.0 to 25.8)		−1.14 (−3.04 to 0.75)	0.24
SCNS-SF34 health system and information	22.3 (21.1 to 23.4)	23.7 (22.5 to 24.9)		−1.46 (−3.13 to 0.21)	0.09
SCNS-SF34 sexuality needs	12.0 (10.9 to13.1)	13.5 (12.4 to 14.7)		−1.56 (−3.11 to −0.01)	<0.05
SCNS-SF34 patient care and support	17.5 (16.5 to 18.6)	19.3 (18.1 to 20.4)		−1.74 (−3.31 to −0.16)	0.03
SCNS-SF34 physical and daily living	27.3 (25.7 to 28.8)	30.0 (28.5 to 31.6)		−2.8 (−5.0 to −0.6)	0.01

CASE-Cancer=Communication and Attitudinal Self-Efficacy scale for cancer; FACT-G=Functional Assessment of Cancer Therapy—General; MSAS=Memorial Symptom Assessment Scale; SCNS-SF34=Supportive Care Needs Survey Short-Form; STAI=State-Trait Anxiety Inventory.

* Adjusted for baseline patient reported outcome measure, cycle, age, sex, cancer type, comorbidity, and country.

† Primary outcome.

Separate analyses for MSAS sub-domains (table 3) showed that mean MSAS global distress index and MSAS psychological scores decreased in both groups once chemotherapy started. However, they were consistently lower in the intervention group during the trial. Least squares mean was 0.46 in the intervention group compared with 0.67 in the control group, giving a difference of −0.21 (−0.27 to −0.16; P<0.001) for MSAS global distress index (effect size 0.42). For MSAS psychological, the mean difference was −0.16 (−0.23 to −0.10; P<0.001) and the effect size was 0.27. By contrast, the MSAS physical score rose during chemotherapy but less so in the intervention arm (0.33 *v* 0.54, with a difference of −0.21 (−0.26 to −0.17; P<0.001) in favour of the intervention and an effect size of 0.58.

In the adjusted analyses, both country and cancer type were related to patient reported outcome measure scores. MSAS scores were generally lower in participants with Hodgkin’s disease or non-Hodgkin’s lymphoma compared with breast or colorectal cancer. Moreover, MSAS scores in UK participants tended to be similar to those from Norway and Ireland, whereas scores from Austria and Greece were significantly lower than those from the UK. However, subgroup analyses by country and type of cancer showed that although benefits were seen in all groups, the greatest benefits of the intervention were in participants with breast cancer, Hodgkin’s disease, or non-Hodgkin’s lymphoma in Austria, Ireland, or the UK.

### Secondary outcomes

FACT-G scores were higher in the intervention group than the control group across all cycles (table 3) (mean difference 4.06, 95% confidence interval 2.65 to 5.46; P<0.001). Similarly, we found a statistically significant between group mean difference in favour of the intervention group on FACT-G physical (mean difference 1.75, 1.25 to 2.25; P<0.001) and functional (1.61, 1.00 to 2.22; P<0.001) domains. The between group mean differences for the FACT-G emotional and social domains were not statistically significant.

We found statistically significant between group differences for mean scores for STAI-R trait anxiety (table 3) (−1.15, –1.90 to –0.41; P=0.003) and STAI-R state anxiety (–1.13, –2.06 to –0.20; P=0.02) in favour of the intervention group. The intervention group reported greater self-care self-efficacy than the control group (0.85, 0.19 to 1.50; P=0.01).

Supportive care needs tended to be lower for most SCNS-SF34 domains in the intervention group (table 3). Psychological needs followed the same trend as MSAS, with a decrease once chemotherapy started and a larger decrease in the intervention group. The largest observed benefits in the intervention group were for sexuality needs (−1.56, −3.11 to −0.01; P<0.05), patient care and support needs (−1.74, −3.31 to −0.16; P=0.03), and physical and daily living needs (−2.8, −5.0 to −0.6; P=0.01). We found no statistically significant differences for the health system and information needs domain.

CASE-Cancer scores were higher in the intervention group than in the control group (mean difference 0.81, 0.19 to 1.43; P=0.01). The WLQ was completed by the 251 patients who were employed, and no significant differences were noted.

### Adherence to the intervention and alert outcomes

DCTAQ adherence was high for the intervention group at 76.9% (33 156 DCTAQs completed; 43 118 DCTAQs available). Of these, 3456 generated a red alert and 3746 an amber alert. Some alerts were “linked,” resulting in 3389 (10.2%) linked red alerts and 3649 (11.0%) linked amber alerts. Similarly, clinicians showed a high adherence rate in terms of dealing with ASyMS alerts within specified timeframes (95% of amber alerts and 85% of red alerts) (table 4). Most amber alerts were managed by using self-care advice (n=2241; 60.6%) and acute oncology referral (n=507; 13.7%). For red alerts, self-care (n=1658; 48.0%) and acute oncology referral (n=1207; 35.0%) were also the two most common alert outcomes (table 5).

**Table 4:** Clinicians’ adherence to time when handling alerts

Type of alert	No of alerts generated	No of linked alerts	No (%) of linked alerts viewed within timescale*
Red alert†	3456	3389	2883 (85.1)
Amber alert‡	3746	3649	3467 (95.0)
Overall	7202	7038	6350 (90.2)

* Time was measured from when first linked alert arrived at server to time when clinician first viewed alert.

† Target time was 30 minutes.

‡ Target time was 8 hours.

**Table 5:** Clinical responses following Advanced Symptom Management System (ASyMS) alerts

Outcome	No of amber alerts	No of red alerts
Self-care	2241	1658
Acute oncology referral	507	1207
Patient not contacted	305	0
Other	317	257
Unable to contact patient; amber alert closed	150	-
No intervention recorded	60	178
Out of hours services	38	18
Emergency services referral	25	77
General practitioner referral	27	29
Hospital referral: clinic review	7	11
Hospital referral: general ward	6	5
Hospital referral: acute oncology referral	5	5
Hospital referral: emergency department	3	4
Hospital referral: specialist	3	2
District nurse referral	2	1
Palliative care team: hospital	2	1
Total*	3698	3453

* Totals are greater than number of alerts as healthcare professionals could select more than one outcome.

### Adverse events

Adverse events were balanced across study arms. Three deaths occurred in each group. Neutropenic events were higher in the intervention group (125 (64%) *v* 71 (36%)). This was expected, as remote monitoring sought to identify these. The numbers of planned (34 *v* 38) and unplanned hospital admissions (120 *v* 109) were similar in the two groups (table 6). No ASyMS device related incidents were reported.

**Table 6:** Adverse events. Values are numbers (percentages)

Adverse event	All patients	Intervention group	Normal care group
Death*	6	3 (50)	3 (50)
Neutropenic sepsis event†	196	125 (64)	71 (36)
Planned hospital admission†	72	34 (47)	38 (53)
Unplanned hospital admission†	229	120 (52)	109 (48)

* Collected in Promasys.

† Collected in case note reviews.

## Discussion

To our knowledge, this trial is the first to evaluate the efficacy of real time remote monitoring of chemotherapy toxicity across multiple countries and to focus mainly on people being treated with curative intent. Overall, symptom burden in the intervention group was effectively controlled, remaining at pre-treatment levels throughout the trial. Similar findings have been reported previously; however, these earlier studies were conducted in a single country and healthcare system.^10^ ^11^ ^12^ ^13^

The greatest improvements were seen in patients with breast cancer, Hodgkin’s disease, or non-Hodgkin’s lymphoma in Austria, Ireland, and the UK. Reasons for differences are unclear, but because ASyMS was developed in the UK it may work best in similar healthcare systems. However, why symptom burden was higher in Norwegian participants is not clear, although it may be because the number of patients from Norway was insufficient to show any difference. eSMART, like other studies,^11^ ^12^ found changes in symptom burden over consecutive chemotherapy cycles. Our findings suggest that benefits of remote monitoring begin within the first three cycles of treatment and are sustained over time, indicating that systems such as ASyMS should be implemented at the start of treatment.

Patients in the intervention group attributed improvements when using ASyMS in terms of enabling real time reporting, the embedded alert algorithms, and direct links to their hospital and oncology team, all of which facilitated timely access to quality care. Similarly, clinicians’ experiences indicated that clinical algorithms and real time symptom reporting enabled provision of targeted and more effective interventions for symptoms. Forthcoming publications will report on patients’ and clinicians’ experiences of the intervention in more detail.

We considered, by convention, that the larger the Cohen’s effect size beyond the 0.25 cut-off point the stronger the indication is for a clinically important change in our primary outcome between intervention and control. From the perspective of clinical importance, the mean between group difference of 0.15 in total MSAS scores translated into an effect size of 0.5, which is considered “medium” according to Cohen’s criteria.^39^ This finding is promising and further substantiates our statistically significant results, pointing to a positive, clinical impact on symptom experience when self-reported symptoms were remotely monitored via ASyMS. Our Cohen’s effect sizes ranged between 0.42 and 0.58 and so are either comparable to or larger than previously reported effect sizes.^36^ ^37^ In a randomised controlled trial testing a symptom monitoring app during neoadjuvant chemotherapy for breast cancer, Fjell et al reported an effect size of 0.26 for between group differences in total MSAS (and 0.34 for MSAS global distress index scores) two weeks after treatment completion.^40^ Effect sizes of 0.01-0.21 for between group differences in MSAS global distress index were reported in a randomised controlled trial of computerised patient reported symptom monitoring during one to four weeks of radiotherapy.^41^

eSMART showed significant improvements in anxiety, health related quality of life, self-efficacy, and supportive care needs in the intervention group. The improvements in health related quality of life are consistent with findings from recent trials using remote monitoring systems in chemotherapy care.^42^ Although Basch et al reported similar results using the EuroQol five dimension (EQ-5D) questionnaire, the dimensions are assessed by a single item.^12^ We used a validated patient reported outcome measure that comprehensively assessed each of these outcomes and provides more detailed findings about the impact of remote monitoring systems not only on health related quality of life but also on self-efficacy and supportive care needs. Our findings suggest that ASyMS has a broader impact, but further analyses using techniques such as parallel process growth modelling are needed to evaluate concurrent changes in symptom burden and health related quality of life outcomes.

eSMART is the first randomised controlled trial in people with cancer to assess the effect of remote symptom monitoring on anxiety by using a validated patient reported outcome measure. ASyMS was associated with statistically significant improvements in anxiety. Our findings may differ from relatively small post-intervention effects on psychological symptoms reported in literature pertinent to routine use of patient reported outcome measures^43^; however, they are consistent with earlier studies of ASyMS in which participants reported feelings of enhanced safety and reassurance.^20^ ^21^ ^23^ ^44^ Anxiety is the most common psychological symptom after a diagnosis of cancer and is higher among people with cancer than in the general population,^45^ ^46^ so these effects may benefit a large proportion of oncology patients; however, this needs to be confirmed in future studies.

Patients using ASyMS reported a statistically significant improvement in self-efficacy, which is consistent with results from the eRAPID trial.^11^ Daily symptom reporting with real time clinician support for moderate-severe symptoms and provision of tailored self-care information are novel features of ASyMS and are likely to promote greater self-efficacy. Other remote monitoring systems that assess symptoms less frequently—for example, weekly^12^— may have limited potential to encourage patients to learn self-management strategies. Likewise, providing real time support from clinicians may engender a greater sense of control. Although other systems integrate symptom reports with patients’ health records, they do not alert clinicians to contact patients within a specified time. Instead, the onus is on patients to contact clinicians when symptoms occur. Recent research on neutropenic sepsis showed that patients are reluctant to report symptoms in a timely manner.^7^

eSMART shows that ASyMS can reduce supportive care needs in several domains during chemotherapy, including psychological, physical, and daily living needs, patient care and support needs, and sexuality needs. The observed reductions may be partially explained by the significant reductions in anxiety and physical symptoms experienced by the intervention group. Daily reporting and feedback from clinicians may have been associated with the receipt of advice on managing physical side effects, with a concomitant decrease in physical and daily living needs. This enhanced symptom support may have resulted in patients perceiving that their clinicians were more sensitive to their needs and decreased their need for supportive care.

Needs relating to sexuality are often overlooked during treatment,^47^ ^48^ so the comprehensive e-library of useful resources, with information on sexual wellbeing, may have been an easily accessible information source. Furthermore, because patients using ASyMS had reduced symptom burden and anxiety, and better health related quality of life, they may have been more inclined to engage in sexual activity.

Evaluating work limitations was an important component of eSMART given the impact of cancer treatment on employment.^49^ ^50^ Work limitation scores were not statistically significantly different between the two groups. This finding is not consistent with a previous study that reported significant work limitations in patients undergoing curative chemotherapy.^51^ However, our results should be interpreted with caution, as only participants who were working completed this patient reported outcome measure.

Patients’ and clinicians’ adherence to the intervention was very good and compares favourably with similar studies that report slightly lower adherence rates,^11^ especially given that eSMART required daily assessments, which some people may consider onerous.

Importantly, our randomised controlled trial found that the safety of ASyMS was satisfactory, with no device related incidents reported. Adverse events were balanced between the two groups, each with three deaths and similar rates of hospital admission. This is not surprising, however, as hospital admission rates in this population are relatively low. The incidence of neutropenic events was higher in the intervention arm, but this was expected as greater identification was the intervention’s intent.

Approximately 30% of patients approached declined participation, with 27% not giving a reason. Those who did mainly cited the psychological impact of diagnosis and limited confidence with technology. Our take-up rate is similar to that in comparable studies that recruited a mix of participants being treated with curative and palliative intent and higher than in those that recruited patients with advanced disease.^11^ ^12^ Anecdotally, clinicians also reported that the intensity of the study at a time when people were feeling stressed about starting treatment was a common concern of those who declined participation. However, the high patient adherence rates seen in this trial serve as a positive indicator of the usability and adoption of ASyMS by patients within the context of their chemotherapy treatment.

### Strengths and limitations of study

This is the largest trial to date of remote monitoring of symptoms during chemotherapy for cancers being treated with curative intent. Notable strengths of this study include its robust randomised controlled trial design, fully powered sample size, longitudinal assessments of symptoms and outcomes, and multi-diagnosis, multisite, and multi-country deployment. Our study, therefore, avoided many limitations of previous studies of remote monitoring that tended to evaluate deployment at single sites and to recruit patients from a single diagnostic group. Our attrition rate was considerably lower than expected and comparable to those in similar studies,^11^ ^12^ demonstrating external validity and high acceptability of the intervention to patients and clinicians.

Our study does, however, have limitations. Firstly, almost three quarters of participants had breast cancer and were female; however, this is a common limitation of supportive cancer care research and reflects the high incidence of breast cancer in Europe. Careful consideration was given before clinical sites were permitted to recruit greater numbers of patients with breast cancer than originally intended, but we judged it more important that the study achieved power than that the proposed diagnostic breakdown was achieved without sufficient power. We recruited participants with Hodgkin’s disease, non-Hodgkin’s lymphoma, and colorectal cancer, so we have evidence that ASyMS could be beneficial in these populations as well. However, with a small number of participants with Hodgkin’s disease or non-Hodgkin’s lymphoma, a larger study is warranted. Seven patients were excluded after randomisation, but associated bias was reduced as numbers were equally distributed between the study groups. Although patients and clinicians were aware of the allocation, trial statisticians were blinded throughout the analysis. Finally, we encountered technical challenges across all sites owing to the connectivity of ASyMS SIM cards, meaning that patients using ASyMS reverted to standard care for approximately two weeks to ensure patient safety while this technical problem was resolved. Although technical testing indicated that this was not related to the ASyMS device, it may have affected overall eSMART results.

### Implications for clinicians and for policy

The results of eSMART support the use of remote symptom monitoring in routine care for patients treated with curative intent. When combined with findings of comparable randomised controlled trials,^10^ ^12^ ^52^ these results support the incorporation of remote symptom monitoring technologies into evidence based guidelines on symptom management in people with cancer. Governments and health organisations are increasingly responding to the rapidly evolving digital health landscape to provide optimal services and care—even more so in response to the covid-19 pandemic—and they recognise ways in which these technologies can disrupt, and are disrupting, the status quo.^53^ Many government policies prioritise empowerment of citizens, enhanced self-management, and digitally enabled access to services.^54^ Our findings suggest that an evidence based remote monitoring intervention, such as ASyMS, has potential for implementation into routine care to make a meaningful difference to people with cancer.

### Conclusion

The results of eSMART suggest that ASyMS is an effective intervention for reducing symptom burden and improving health related quality of life during adjuvant chemotherapy across a range of cancers. Use of ASyMS was associated with significant reductions in anxiety and improvements in several supportive care needs and self-efficacy domains. Moreover, results were consistent across five European countries, although perhaps with greater impact in Austria, the Republic of Ireland, and the UK. Our success in implementing ASyMS across several diverse health systems suggests that the system can be easily scaled up and adapted for use in various international settings.

Improving symptom management by using remote monitoring systems such as ASyMS is essential. Future research should combine artificial intelligence with the use of real world data to develop predictive, personalised, and targeted interventions. These approaches are likely to lead to improvements in patients’ outcomes and efficiencies in care. Evaluation of the efficacy of remote symptom monitoring systems such as ASyMS for other treatment modalities (for example, targeted therapies) is needed. The ultimate vision is to have a multimodal seamless system of remote symptom monitoring used from the start of treatment and through survivorship.

Our findings are relevant in the context of the covid-19 pandemic. The cancer community faces unprecedented challenges in delivering chemotherapy,^55^ but ASyMS can provide a safe, secure, and “real time” system that optimises symptom management and supports patients to remain at home. Essentially, the system can expedite informed and appropriate patient triage and enable clinicians to care for multiple patients in real time, using digital lines of communication to deliver high quality and safe care at a distance.

## What is already known on this topic

Effective symptom monitoring and management is essential during chemotherapy for cancer
Current approaches for reporting symptoms rely on patients’ retrospective recall and self-identification of severe symptoms to prompt contact with their clinicians
Digital remote monitoring interventions to support patients during chemotherapy are available, but very few were evaluated in cancers being treated with curative intent

## What this study adds

ASyMS is an effective intervention for reducing symptom burden and improving quality of life during adjuvant chemotherapy for people with breast cancer, colorectal cancer, Hodgkin’s disease, and non-Hodgkin’s lymphoma
ASyMS has a positive effect on a range of additional important outcomes for patients during chemotherapy, including anxiety and self-efficacy
Digital solutions for remote monitoring and managing of chemotherapy symptoms can be delivered across multiple sites in European countries with diverse healthcare systems

## Notes

Contributors: RM, LM, GK, NK, ER, JA, PF, EP, EF, PF, AG, PMcC, GB, CM, DO, AF, SK, and PTD obtained funding for the research. All authors contributed to either the design or conduct of the study. NDS did the statistical analyses, supervised by PTD. RM, PTD, and LM produced the first draft of this manuscript, and all authors reviewed, edited, and approved the final version. The corresponding author attests that all listed authors meet authorship criteria and that no others meeting the criteria were omitted. RM and PTD are the guarantors.

Data sharing; The study protocol was published previously. Data will not be released to any external parties for the first 12 months following publication of this manuscript. After this time, requests for access to de-identified individual participant data that underlie the results reported in this article should be addressed to the corresponding author and should include clear and specified plans of how the data are to be used. All proposals for data use will need approval of the eSMART consortium partner leads before any data are released.

The lead authors affirm that the manuscript is an honest, accurate, and transparent account of the study being reported; that no important aspects of the study have been omitted; and that any discrepancies from the study as planned (and, if relevant, registered) have been explained.

Dissemination to participants and related patient and public communities: The European Cancer Patient Coalition will disseminate the findings of eSMART to relevant patient and public communities across Europe; however, dissemination of the results to the eSMART participants is not possible.

Provenance and peer review: Not commissioned; externally peer reviewed.
